# HP1a-mediated heterochromatin formation inhibits high dietary sugar-induced tumor progression

**DOI:** 10.1038/s41419-021-04414-z

**Published:** 2021-12-06

**Authors:** Che-Wei Chang, Yu-Chia Shen, Shian-Jang Yan

**Affiliations:** 1 Institute of Basic Medical Sciences, College of Medicine, National Cheng Kung University, No. 1, University Road, Tainan City, Taiwan; 2 Department of Physiology, College of Medicine, National Cheng Kung University, No. 1, University Road, Tainan City, Taiwan

**Keywords:** Cancer metabolism, Chromatin structure

## Abstract

High dietary sugar (HDS) is a modern dietary concern that involves excessive consumption of carbohydrates and added sugars, and increases the risk of metabolic disorders and associated cancers. However, epigenetic mechanisms by which HDS induces tumor progression remain unclear. Here, we investigate the role of heterochromatin, an important yet poorly understood part of the epigenome, in HDS-induced tumor progression of *Drosophila* Ras/Src and Ras/scrib tumor systems. We found that increased heterochromatin formation with overexpression of heterochromatin protein 1a (HP1a), specifically in tumor cells, not only decreases HDS-induced tumor growth/burden but also drastically improves survival of *Drosophila* with HDS and Ras/Src or Ras/scrib tumors. Moreover, HDS reduces heterochromatin levels in tumor cells. Mechanistically, we demonstrated that increased heterochromatin formation decreases wingless (wg) and Hippo (Hpo) signaling, thereby promoting apoptosis, via inhibition of Yorkie (Yki) nuclear accumulation and upregulation of apoptotic genes, and reduces DNA damage in tumor cells under HDS. Taken together, our work identified a novel epigenetic mechanism by which HP1a-mediated heterochromatin formation suppresses HDS-induced tumor progression likely by decreasing wingless and Hippo signaling, increasing apoptosis, and maintaining genome stability. Our model explains that the molecular, cellular, and organismal aspects of HDS-aggravated tumor progression are dependent on heterochromatin formation, and highlights heterochromatin as a therapeutic target for cancers associated with HDS-induced metabolic disorders.

## Introduction

High dietary sugar (HDS), a modern dietary concern, involves high intake of total sugars, including added sugars and free sugars. HDS is associated with an increased risk of metabolic disorders (e.g., type 2 diabetes) and associated cancers, which are two leading causes of death worldwide [1, 2]. HDS increases tumor growth in a variety of animal models [3]. Cancer cells tend to generate energy via the “Warburg effect”-increased glucose consumption and anaerobic glycolysis [4–6]. Cancer cells may alter their growth signaling and metabolic states under HDS by upregulating the wingless/Wnt pathway to increase cancer growth [7] and promoting glycolysis [8, 9], respectively. Moreover, HDS can induce nucleotide imbalance, DNA damage, and oncogene KRAS mutation in cancer cells [10]. However, the ways in which HDS-associated epigenetic mechanisms induce tumor progression remain poorly understood.

Heterochromatin, a key architectural chromatin structure that is more compact than euchromatin, is crucial for organizing the eukaryotic genome supporting important biological functions, such as gene regulation and genome stability [11]. Histone post-translational modifications, particularly di/tri-methylation of histone 3 and lysine 9 (H3K9me2/me3), contribute to heterochromatin formation and are, therefore, heterochromatin markers. Heterochromatin protein 1a (HP1a), a key molecule in heterochromatin formation, is bound to H3K9me2/me3 sites and assists in formation of condensed heterochromatin structure, which leads to reduced gene expression by making genes less accessible to the binding of transcription factors. Heterochromatin formation also ensures proper chromosome segregation during cell division and maintains genome stability [12].

Recent evidence has demonstrated that epigenetic lesions, such as heterochromatin instability, and the dysregulation of chromatin structure state lead to cancer [13]. Induction of heterochromatin formation through HP1 protein binding, which suppresses gene expression, has been identified as a potential key control point in cancer development [14]. HP1α is downregulated in numerous human cancers [15]; however, the role of HP1-mediated heterochromatin formation in HDS-induced cancer development is unclear.

Here, we use *Drosophila* Ras/Src and Ras/scrib cancer models to investigate the role of epigenetic regulation in HDS-induced cancer development. Overexpression or mutation of the oncogene *rat sarcoma* (*ras*) has been found in 20–30% of all human cancers [16]. Reliable in vivo cancer models with consistent overexpression of a constitutively active form of Ras and loss of either tumor suppressor *C-terminal Src kinase* (*csk*) or *scribble* (*scrib*) have been generated in the eye discs of *Drosophila* larvae [17, 18]. Importantly, the nucleotide and protein sequences of *ras*, *csk* and *scrib* are highly conserved from the fly to humans. Moreover, it is noteworthy that more than 50% of human disease-causing genes, including oncogenes and tumor suppressor genes, have an orthologue in *D. melanogaster* [19]. Thus, these *Drosophila* cancers of the imaginal discs are models for the study of human epithelial cancers, and have provided an excellent, genetically tractable system through which to dissect the mechanisms of tumor progression in vivo [20]. In this study, we investigated the role of HP1a-mediated heterochromatin formation in HDS-induced tumor progression of *Drosophila* Ras/Src and Ras/scrib tumor systems. By building an HDS-induced tumor system in which heterochromatin levels are manipulated through HP1a overexpression/knockdown/mutation, we found that HP1a-mediated heterochromatin formation reduces HDS-induced developmental delay and lethality in both Ras/Src and Ras/scrib tumor-bearing flies. Furthermore, HP1a-mediated heterochromatin formation reduces tumor progression by inhibiting Yki nuclear accumulation and activating the apoptotic pathway, likely via upregulating *Rpr* and *Wts*, two apoptotic genes. Our study characterizes the molecular and cellular basis of heterochromatin formation in HDS-induced tumor progression that occurs likely via regulation of the wingless/dWnt, Hippo, apoptosis and genome maintenance pathways. Altogether, this study offers novel epigenetic insights into the intricate pathology of HDS-induced tumor progression via downregulation of HP1a-mediated heterochromatin formation.

## Materials and methods

### Fly strains

The following fly strains were gifts from Dr. Ross Cagan at Icahn School of Medicine (Mount Sinai Hospital, New York, NY, USA): [1] *ey (3.5)-FLP1; act* > *y* + *>gal4, UAS-GFP; FRT82B, tub-gal80*; [2] *UAS-lacZ; FRT82B* and [3] *UAS-ras*^*G12V*^*; FRT82B, csk*^*Q156Stop*^*/TM6b*. The following fly strain was a gift from Dr. José Carlos Pastor-Pareja at Tsinghua University, China: [5] *UAS-ras1*^*G12V*^*; FRT82B, scrib*^*1*^*/TM6b*; and another four strains, including [6] *UAS-ras*^*G12V*^*, HP1a-RNAi; FRT82B, csk*^*Q156Stop*^, [7] *UAS-ras*^*G12V*^*, UAS-HP1a; FRT82B, csk*^*Q156Stop*^, [8] *UAS-ras*^*G12V*^*, UAS-HP1a; FRT82B, scrib*^*1*^*/TM6b*, and [9] *UAS-ras*^*G12V*^*, UAS-HP1a-RNAi; FRT82B, scrib*^*1*^*/TM6b* were constructed by chromosome recombination. Virgins of strain *ey (3.5)-FLP1; act* > *y* + *>gal4, UAS-GFP; FRT82B, tub-gal80* were crossed with flies of the above strains [2] through [9] to generate tumor-bearing flies with different heterochromatin levels. Other strains that were used, including *UAS-HP1a*, *UAS-HP1a-RNAi* (VDRC stock 31995, used extensively in this study unless specified; BDSC stock 33400 and 36792); *w*^*m4*^, *Oregon R*, and *w*^*1118*^ were obtained from Bloomington Drosophila Stock Center (Indiana University, Bloomington, IA, USA). The *tub-geneswitch (GS)-Gal4* strain was a kind gift from Dr. Dirk Bohmann at the University of Rochester (Rochester, NY, USA). The *UAS-lacZ; eyeless (ey)-Gal4* strain was a kind gift from Dr. Anna C.C. Jang at National Cheng Kung University.

### Fly culture

Flies were cultured at 25 °C unless otherwise noted. Culture was carried out in Bloomington semi-defined medium, as described by the Bloomington Stock Center, with modifications. To examine diet-induced effects on metabolism, flies were fed the control diet (0.15 M sucrose) or high-calorie food. The high-calorie diet containing high sugar (0.75 M sucrose) was the experimental condition we used to study HDS-induced tumor progression, and was modified from Musselman, L. P. et al. [21].

Developmental delay was defined as longer time to pupation for the 50th percentile of flies in the experimental group compared to when the 50th percentile of control flies reached pupation. The survival rate was defined as the percentage of flies attaining eclosion.

### Western blotting

After mating for one day, adult flies were collected and separated by sex. The heads of 30 flies were dissected out and mixed with beads and RIPA buffer containing a protease inhibitor cocktail. Protein concentration was assessed by Bradford assay (Bio-Rad, USA).

Protein lysate was separated on a polyacrylamide gel and then transferred to Immobilon^TM^ membrane. The blotting membrane was probed with primary antibodies: HP1a (C1A9; Developmental Studies Hybridoma Bank), H3K9me2 (07-212; Upstate Biotechnology), H3 (05-928; Millipore), or Syntaxin (8C3; Developmental Studies Hybridoma Bank). Blots were probed with anti-rabbit or anti-mouse secondary antibodies (Invitrogen, USA) and signal was detected using enhanced ECL chemiluminescence. Image J software was used for western blot quantification.

### Immunofluorescence

Larval eye discs were dissected out and rinsed in PBS. They were then fixed for 10 min with 4% paraformaldehyde (PFA) in PBS, washed in PBST (PBS containing 0.1% Triton X-100) three times, and incubated with primary antibodies, followed by washing and incubation with secondary antibodies. The tissues were incubated with mouse monoclonal anti-HP1a (C1A9, 1:200; Developmental Studies Hybridoma Bank) and/or mouse monoclonal anti-β-galactosidase (JIE7, 1:200; Developmental Studies Hybridoma Bank), mouse monoclonal anti-wingless (4D4, 1:250; Developmental Studies Hybridoma Bank), and/or rabbit anti-H3K9me2 (07-212, 1:200; Upstate Biotechnology) and/or rabbit anti-phospho-histone 3 (06-570, 1:250; Millipore) and/or rabbit anti-Yki [22] (1:500, a gift from Dr. Jenn-Yah Yu at National Yang Ming Chiao Tung University) in PBST with normal goat serum (NGS). Eye discs then were incubated with goat anti-rabbit/anti-mouse conjugated to Alexa Fluor-594 (Thermo Fisher Scientific, USA) or goat anti-rabbit/anti-mouse conjugated to Alexa Fluor-660 (Thermo Fisher Scientific). Discs were further mounted with a Vectashield dye (Vector Laboratories, USA) and images were taken with a confocal microscope (Carl Zeiss LSM780, Instrument Development Center, National Cheng Kung University, Tainan, Taiwan). All confocal images were minimally processed by the Zeiss ZEN imaging software, and image processing was applied equally across the entire image and to controls.

### TUNEL assay

Larval eyes were dissected out, rinsed in PBS and then fixed in 4% PFA for 15 min at room temperature. After being washed in PBS four times, they were incubated with 100 mM sodium citrate/0.1% Triton X-100 at 65 °C for 30 min on a shaker at 200 r.p.m. for permeabilization. They were then washed twice with 3% BSA in PBS and incubated with 1× TdT reaction buffer at 37 °C for 10 min (Click-iT™ Plus TUNEL Assay for In Situ Apoptosis Detection, Thermo Fisher Scientific, USA). The previous substrate was removed, and discs were incubated with the TdT reaction cocktail at 37 °C for 60 min. The tissues were again washed twice with 3% BSA in PBS and then incubated with the Click-iT™ Plus TUNEL Supermix at 37 °C for 30 min. Tissues were mounted with a Vectashield dye (Vector Laboratories) and images were taken with a confocal microscope (Carl Zeiss LSM780, Instrument Development Center, National Cheng Kung University, Tainan, Taiwan).

### Geneswitch inducible system

To induce expression of specific genes, adult flies were cultured with food containing 300 μM RU486 or solvent (EtOH)-only as a control. After 4 days in culture with the fly food, flies were homogenized, and cells were lysed for RNA extraction.

### Eye pigment extraction

The heads of 10 adult flies were dissected and rinsed in PBS, and then homogenized in glass beads with 0.1% HCl in methanol. After centrifugation at 12,000 r.c.f., the supernatant was collected and the emission spectra were assessed at 480 nm using a spectrophotometer.

### Adult eye imaging

To image the external eyes, the heads of 3-day-old female adult flies were removed and put on agarose gel plates after the flies were anesthetized. Images were taken with a stereo fluorescence microscope.

### Quantitative RT-PCR

RNA was extracted from 10 adult flies or 30 heads of adult flies using TRIzol Reagent (Thermo Fisher Scientific) according to the kit instructions. cDNA was converted by MScript RTase (GeneDirex, Taiwan). Quantitative RT-PCR was carried out using Power SYBR Green PCR Mix (Applied Biosystems) and analyzed on a StepOnePlus PCR system. *rpl32* was used as an internal control. The following primers were used:

*rpl32* Forward: 5′-GCTAAGCTGTCGCACAAATG-3′,

*rpl32* Reverse: 5′-GTTCGATCCGTAACCGATGT-3′;

*HP1*a Forward: 5′-CGCAAGGATGAGGAGAAGTCA-3′,

*HP1a* Reverse: 5′-TCCTGAAACGGGAATGGTGTC-3′;

*rpr* Forward: 5′-TCCACTGTGACTCCCGCAAG-3′,

*rpr* Reverse: 5′-GCCAGCAACAAAGAACTAACTCG-3′;

*hid* Forward: 5′-CCAAAACGAAAACGGTCACAAC-3′,

*hid* Reverse: 5′-TCGCTACGACAACTTTACGG-3′;

*Diap1* Forward: 5′-ATAGACACAATGGACAACTCGCT-3′,

*Diap1* Reverse: 5′-CTGAAGTCGAAACTTGACGGC-3′;

*wts* Forward: 5′-ATGACGGCCCTTAATGCCAAA-3′,

*wts* Reverse: 5′-TCCGCCTGGGTATAGGTTCA-3′;

*SUV39* Forward: 5′-CAAGCGGTCGAAAAATAACATGGG-3′,

*SUV39* Reverse: 5′-TGCCTCCAGCTGCTTCTCAAGCT-3′;

*P53* Forward: 5′-CCAAGCTAGAGAATCACAACATCG-3′,

*P53* Reverse: 5′-TCGAGTACATCCAAAGAGACTTGG-3′

### Statistical analysis

All results analyzed and presented reflected data from at least three independent experiments, using PRISM 6 (GraphPad, San Diego, CA). Results were shown as mean ± SD. The statistical analysis was performed by two-way ANOVA or Student’s *t*-test as specified in the figure legend. The significant level was set as *p* values below 0.05.

## Results

### Heterochromatin formation suppresses HDS-induced developmental delay and lethality of Ras/Src and Ras/scrib tumor-bearing flies

**Fig. 1:**
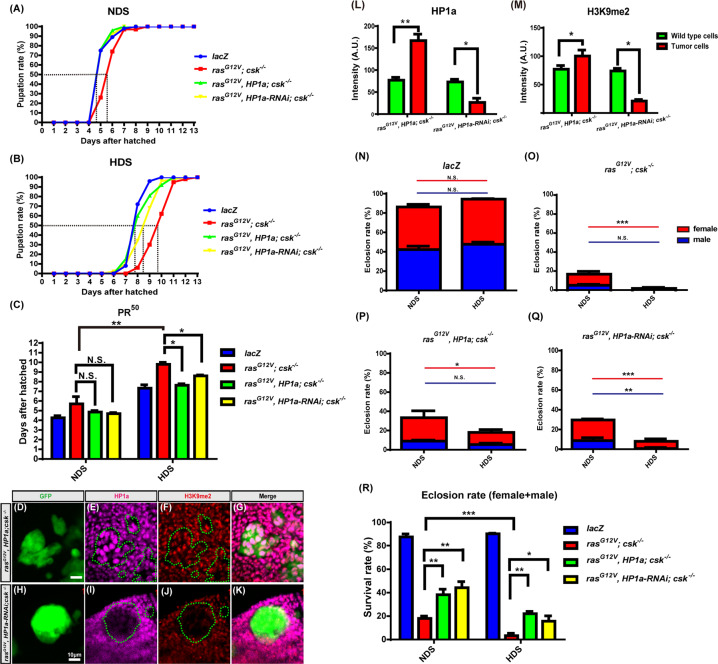
Increased HP1a-mediated heterochromatin formation decreases HDS-induced developmental delay and lethality of Ras/Src tumor-bearing flies. **A**, **B** Pupation rate of animals fed a 0.15 M sucrose diet (NDS) and a 0.75 M sucrose diet (HDS), respectively, with the following genotypes: *lacZ*, and *ras*^*G12V*^*; csk*^*−/−*^ and *ras*^*G12V*^, *HP1a; csk*^*−/−*^ and *ras*^*G12V*^*, HP1a-RNAi; csk*^*−/−*^. Each genotype contains at least 150 flies. **C** Developmental time via 50% pupation rate (PR50) of animals, fed NDS or HDS, that had the following genotypes: *lacZ*, and *ras*^*G12V*^*; csk*^*−/−*^ and *ras*^*G12V*^, *HP1a; csk*^*−/−*^, and *ras*^*G12V*^*, HP1a-RNAi; csk*^*−/−*^. Each genotype contains at least 150 flies. **D**–**K** Third instar eye discs of *ras*^*G12V*^*, HP1a; csk*^*−/−*^ (**D**–**G**) and *ras*^*G12V*^*, HP1a-RNAi; csk*^*−/−*^ (**H**–**K**) flies fed NDS with GFP-labeled tumor cells (green), and HP1a (magenta) and H3K9me2 (red) immunostaining. Scale bar: 10 μm. **L**, **M** Immunofluorescence intensity of HP1a and H3K9me2 in tumor cells compared to that of wild-type cells. All results analyzed and presented reflect data from three independent experiments (*n* = 15). **N**–**Q** Eclosion rate of animals, fed NDS or HDS, with the following genotypes: **N** *lacZ*, **O** *ras*^*G12V*^*; csk*^*−/−*^, **P** *ras*^*G12V*^*, HP1a; csk*^*−/−*^, **Q** *ras*^*G12V*^*, HP1a-RNAi; csk*^*−/−*^ (red line, female; blue line: male). **R** The survival rates of animals, fed NDS or HDS, with the following genotypes: *lacZ*, and *ras*^*G12V*^*; csk*^*−/−*^ and *ras*^*G12V*^, *HP1a; csk*^*−/−*^, and *ras*^*G12V*^*, HP1a-RNAi; csk*^*−/−*^. All results analyzed and presented reflect data from three independent experiments (*n* = 450). Results are shown as mean ± SD. Asterisks indicate statistically significant differences (*p < 0.05; **p < 0.01; ***p < 0.001). GFP green fluorescent protein, NS not significant.

HDS (1.0 M sucrose) has been shown to increase Ras/Src tumor growth/burden in *Drosophila* eye/antenna discs compared to normal dietary sugar (NDS, 0.15 M sucrose) [23]. In our study, we found that HDS at 0.75 M sucrose (hereafter, simply “HDS”) also promotes Ras/Src tumor burden, albeit with reduced lethality compared to 1.0 M sucrose, which causes 100% lethality (data not shown). Compared to NDS, HDS not only increased *ras*^*G12V*^*; csk*^*−/−*^ tumor cell growth but also caused developmental delay of tumor-bearing flies by extending the larval period before pupation. The mortality of Ras/Src tumor-bearing flies fed HDS was dramatically increased compared to non-tumor-bearing flies fed HDS. Importantly, increasing heterochromatin formation by HP1a overexpression, specifically in tumor cells, reversed both developmental delay and mortality of the Ras/Src tumor-bearing flies fed HDS. Flies with *ras*^*G12V*^*, HP1a; csk*^−/−^ tumors showed a developmental timeline similar to control non-tumor-bearing flies fed NDS or HDS (Fig. 1A, B). Under HDS, the time to reach 50% pupation rate (PR^50^), when 50% of the larvae have reached the pupal stage, was increased in *ras*^*G12V*^*; csk*^−/−^ tumor-bearing flies compared to control wild-type flies (Fig. 1B, C). However, HP1a overexpression in *ras*^*G12V*^*, HP1a; csk*^−^^/−^ tumor-bearing flies shortened the timeline to PR^50^ to that observed in control non-tumor-bearing flies (Fig. 1B, C). Interestingly, decreasing heterochromatin formation via HP1a knockdown by expressing HP1a-RNAi in *ras*^*G12V*^*, HP1a-RNAi; csk*^−/−^ animals also reduced the developmental delay in response to HDS compared to controls, although to a lesser extent than that observed in *ras*^*G12V*^*, HP1a; csk*^−^^/−^-expressing flies (Fig. 1B, C). Moreover, we verified that heterochromatin levels, based on expression of heterochromatin markers HP1a and H3K9me2, increased by 120% and 23%, respectively, and specifically in Ras/Src tumor cells overexpressing HP1a, i.e., tumor clones of *ras*^*G12V*^*, HP1a; csk*^−^^/^^−^ flies, but not in surrounding non-tumor cells (Fig. 1D–G). HP1a and H3K9me2 levels were decreased by 65% and 72%, respectively, by HP1a knockdown in the tumor clones of *ras*^*G12V*^*, HP1a-RNAi; csk*^*−/−*^ flies (Fig. 1H–M). Thus, HP1a overexpression and HP1a knockdown specifically in the tumor clones effectively increased and decreased heterochromatin levels, respectively, in the Ras/Src tumor model. Furthermore, the eclosion/survival rate of female flies with HDS and HP1a overexpression of *ras*^*G12V*^*, HP1a; csk*^*−/−*^ was increased by 20% compared to that of *ras*^*G12V*^*; csk*^*−/−*^ tumor-bearing flies fed HDS. Both female and male flies with HDS and HP1a knockdown of *ras*^*G12V*^*, HP1a-RNAi; csk*^*−/−*^ also showed improved eclosion/survival (Fig. 1N–R). These results suggest that increasing heterochromatin formation by HP1a overexpression in tumor cells suppresses HDS-induced developmental delay and lethality in Ras/Src tumor-bearing flies. Interestingly, drastic reduction of heterochromatin in tumor cells also suppresses HDS-induced tumor phenotypes.

To further characterize HDS involvement in cancer development, we assessed whether HDS exacerbated tumor growth in the more severe Ras/scrib tumor system carrying the *ras*^*G12V*^; *scrib*^*−/−*^ genetic modifications [24]. We found that HDS enhanced tumor growth (data not shown), developmental delay, and lethality in *ras*^*G12V*^; *scrib*^*−/−*^ tumor-bearing flies, which showed reduced pupation and no eclosion (Supplement Fig. 1). More importantly, HP1a-mediated heterochromatin formation not only diminished the HDS-induced developmental delay but also increased the pupation rate of flies with either NDS or HDS (Supplement Fig. 1A–D). Consistent with the results from the Ras/Src tumor system, HP1a overexpression and HP1a knockdown, specifically in the tumor clones, effectively increased and decreased heterochromatin levels, respectively, in Ras/scrib tumor-bearing flies models (Supplement Fig. 1E–L). Furthermore, we examined knockdown efficiency of multiple HP1a-RNAi lines, and found that the VDRC 31995 line, which was used extensively in this study, but not the others, effectively reduced the HP1a protein level in *Drosophila* eye discs (Supplement Fig. 2). Therefore, our data demonstrate that heterochromatin formation plays an important role in the suppression of HDS-induced developmental delay and lethality in Ras/scrib tumor-bearing flies.

To further investigate the role of HP1a-mediated heterochromatin formation in HDS-induced tumorigenesis, we constructed two more strains of Ras/Src tumor-bearing flies with *hs-HP1a*, an *HP1a* transgene overexpressing HP1a by basal activity of the hsp70 promoter, with increased HP1a expression by 20% [25], and *HP1*^*04/+*^, *HP1a* heterozygotes of a null-mutation allele, with decreased *HP1a* levels by 50% [26], respectively. Consistently, increasing heterochromatin formation via *hs-HP1a* in *ras*^*G12V*^*, hs-HP1a; csk*^*−/−*^ animals also suppressed tumor growth and improved eclosion/survival of female flies fed HDS (Supplement Fig. 3). Moreover, decreasing heterochromatin formation via *HP1*^*04/+*^ with reducing HP1a levels by half, as in *ras*^*G12V*^*, HP1a*^*+/−*^*; csk*^*−/−*^ animals, with HDS, showed no improvement in tumor growth and eclosion/survival of tumor-bearing flies compared to those of *ras*^*G12V*^*; csk*^*−/−*^ tumor-bearing flies fed HDS (Supplement Fig. 3).

Taken together, these results suggest that heterochromatin levels are critical for HDS-induced tumor progression. Increased heterochromatin formation suppressed HDS-induced tumor phenotypes, including developmental delay and lethality, in two independent in vivo tumor models.

### HDS reduces heterochromatin formation

**Fig. 2:**
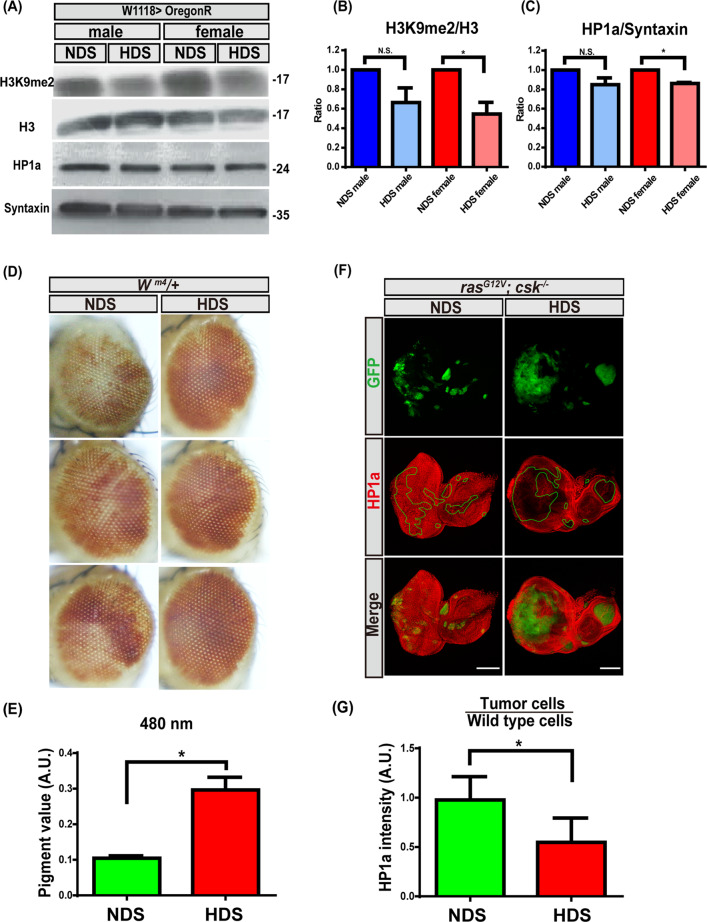
HDS decreases heterochromatin formation. **A** Western blots of protein from the heads of wild type adult flies fed NDS or HDS, using antibodies against H3K9me2, H3, HP1a, or Syntaxin (loading control). **B**, **C** Quantification of **B** H3K9me2 and **C** HP1a levels from western blots in **A**, normalized to H3 and Syntaxin, respectively. All results analyzed and presented reflect data from three independent experiments (*n* = 90). **D** Eyes from adult *w*^*m4*^ outcrossed wild-type *w*^*1118*^ flies fed NDS or HDS for assessment via the PEV assay. **E** Pigmentation data from the eyes pictured in **D**, after pigment was extracted and measured at 480 nm. All results analyzed and presented reflect data from three independent experiments (*n* = 30). **F** Third instar eye discs of *ras*^*G12V*^*; csk*^*−/−*^ tumor-bearing flies fed NDS or HDS, with GFP-labeled tumor cells (green) and HP1a immunostaining (red). Scale bar: 100 μm. **G** Immunofluorescence intensity of HP1a in tumor cells from flies fed HDS compared to those of tumor cells from flies fed NDS. Staining intensity was normalized to that of non-tumor cells in each experimental condition. All results analyzed and presented reflect data from three independent experiments (*n* = 15). Results are shown as mean ± SD. Asterisk indicates statistically significant difference (*p < 0.05). GFP green fluorescent protein, HDS high dietary sugar, NDS normal dietary sugar, NS not significant.

It is unclear whether HDS alters heterochromatin levels in vivo. To explore this question, we first measured the heterochromatin levels by western blot in wild-type flies fed NDS or HDS. We found that HDS led to downregulation of heterochromatin levels in female, but not male, *Drosophila* heads, which included the eyes. Specifically, the levels of H3K9me2 and HP1a proteins, which are markers of binding sites for heterochromatin formation, were downregulated by HDS compared to NDS in female flies (Fig. 2A–C). Furthermore, using the position-effect variegation (PEV) assay, which reveals the levels of heterochromatin in adult *Drosophila* eyes, we found that flies fed HDS had reduced heterochromatin levels compared to those fed NDS, which appeared as less mosaic eye color and more eye pigmentation (Fig. 2D, E). These results suggest that HDS reduces heterochromatin formation.

Next, to determine whether HDS also reduced heterochromatin levels in tumor cells, we measured the HP1a levels via immunostaining in Ras/Src tumor cells from flies fed either NDS or HDS. Indeed, HP1a was reduced by 50% in Ras/Src tumor cells from flies fed HDS compared to those fed NDS (Fig. 2F, G). Thus, our data indicate that HDS reduces heterochromatin formation in tumor cells. Results from both wild-type flies and tumor-bearing flies fed HDS suggest that HDS decreases heterochromatin formation, which may promote tumor progression.

### Increased heterochromatin formation in Ras/Src tumor cells suppresses HDS-induced tumor growth

**Fig. 3:**
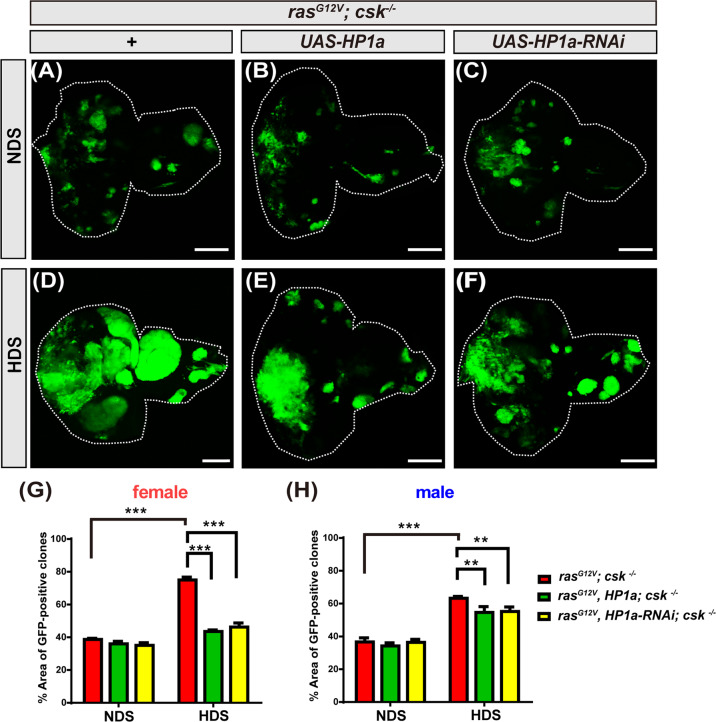
Increased heterochromatin formation in Ras/Src tumor cells suppresses HDS-induced tumor growth. Third instar eye discs of *ras*^*G12V*^*; csk*^*−/−*^ and *ras*^*G12V*^*, HP1a; csk*^*−/−*^ and *ras*^*G12V*^*, HP1a-RNAi; csk*^*−/−*^ flies fed NDS or HDS; tumor cells are labeled with GFP (green). Scale bar: 100 μm. **A**, **D** *ras*^*G12V*^*; csk*^*−/−*^. **B**, **E** *ras*^*G12V*^*, HP1a*; *csk*^*−/−*^. **C**, **F** *ras*^*G12V*^*, HP1a-RNAi*; *csk*^*−/−*^. **G**, **H** Percentage of GFP-positive tumor cells normalized to total eye disc area from *ras*^*G12V*^*; csk*^*−/−*^ and *ras*^*G12V*^*, HP1a; csk*^*−/−*^ and *ras*^*G12V*^*, HP1a-RNAi; csk*^*−/−*^ female or male flies fed NDS or HDS. All results analyzed and presented reflect data from five independent experiments (*n* = 50); results are shown as mean ± SD of individual eye discs. Asterisks indicate statistically significant differences (**p < 0.05; ***p < 0.01). GFP green fluorescent protein, HDS high dietary sugar, NDS normal dietary sugar.

To further determine the cellular mechanisms by which HP1a-mediated heterochromatin formation suppresses developmental delay and lethality of HDS-fed Ras/Src and Ras/scrib tumor-bearing flies, we asked whether increased heterochromatin formation, specifically in Ras/Src tumor cells, could suppress HDS-induced tumor growth. Indeed, increased heterochromatin formation induced by HP1a overexpression reduced tumor growth in *ras*^*G12V*^*, HP1a; csk*^*−/−*^ in both female and male flies that were fed HDS, with more drastic reduction of tumor size in females, while HP1a knockdown also reduced tumor growth in *ras*^*G12V*^*, HP1a-RNAi; csk*^*−/−*^ flies (Fig. 3). These data indicate that heterochromatin formation plays a critical role in suppressing HDS-induced growth of tumor cells.

### Increased heterochromatin formation reduces proliferation of tumor cells from flies fed NDS

**Fig. 4:**
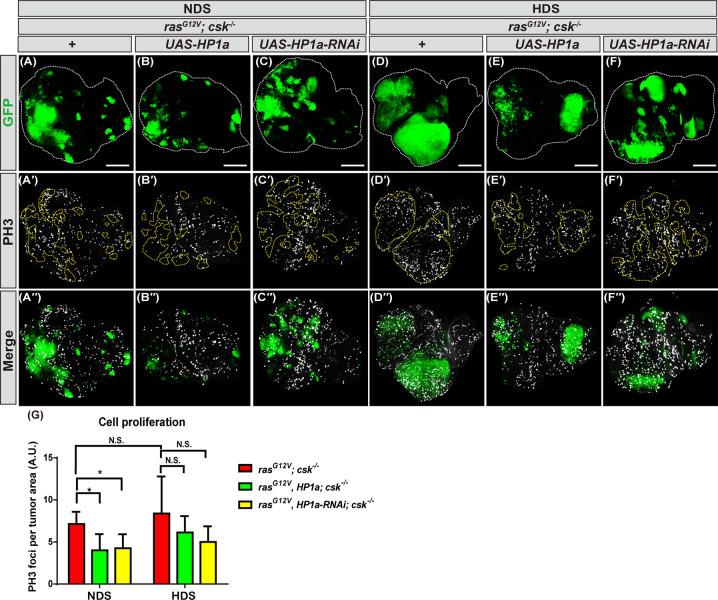
Increased heterochromatin formation reduces proliferation of tumor cells from flies fed NDS. Third instar eye discs of *ras*^*G12V*^*; csk*^*−/−*^ and *ras*^*G12V*^*, HP1a; csk*^*−/−*^ and *ras*^*G12V*^*, HP1a-RNAi; csk*^*−/−*^ flies fed NDS or HDS with GFP-labeled tumor cells (green) and PH3 immunostaining (gray). Scale bar: 100 μm. **A**–**A′′, D**–**D′′** *ras*^*G12V*^*; csk*^*−/−*^. **B**–**B′′, E**–**E′′** *ras*^*G12V*^*, HP1a*; *csk*^*−/−*^. **C**–**C′′, F**–**F′′** *ras*^*G12V*^*, HP1a-RNAi*; *csk*^*−/−*^. **G** Fluorescent quantification of PH3 puncta in tumor cells from *ras*^*G12V*^*; csk*^*−/−*^ and *ras*^*G12V*^*, HP1a; csk*^*−/−*^ and *ras*^*G12V*^*, HP1a-RNAi; csk*^*−/−*^ flies fed NDS or HDS. All results analyzed and presented reflect data from three independent experiments (*n* = 15). Results are shown as mean ± SD of individual eye discs. Asterisk indicates a statistically significant difference (*p < 0.05). GFP green fluorescent protein, HDS high dietary sugar, NDS normal dietary sugar, NS not significant.

To further investigate the mechanisms by which increased heterochromatin formation suppresses HDS-induced Ras/Src tumor growth, we first tested whether heterochromatin formation decreases HDS-induced tumor cell proliferation using phosphohistone-H3 (PH3) staining, which marks mitotic cells [27]. Ras/Src tumor cells with HP1a overexpression or knockdown from flies fed NDS showed decreased mitosis (Fig. 4A–C′′). Interestingly, HP1a overexpression or knockdown showed a non-significant trend toward reduction of mitosis in tumor cells under HDS at the late third instar stage (Fig. 4D–F′′, G). Together, these results suggest that increased heterochromatin formation suppresses tumor cell proliferation in flies fed NDS, but less so in those fed HDS.

### Increased heterochromatin formation increases apoptosis by in tumor cells under HDS

**Fig. 5:**
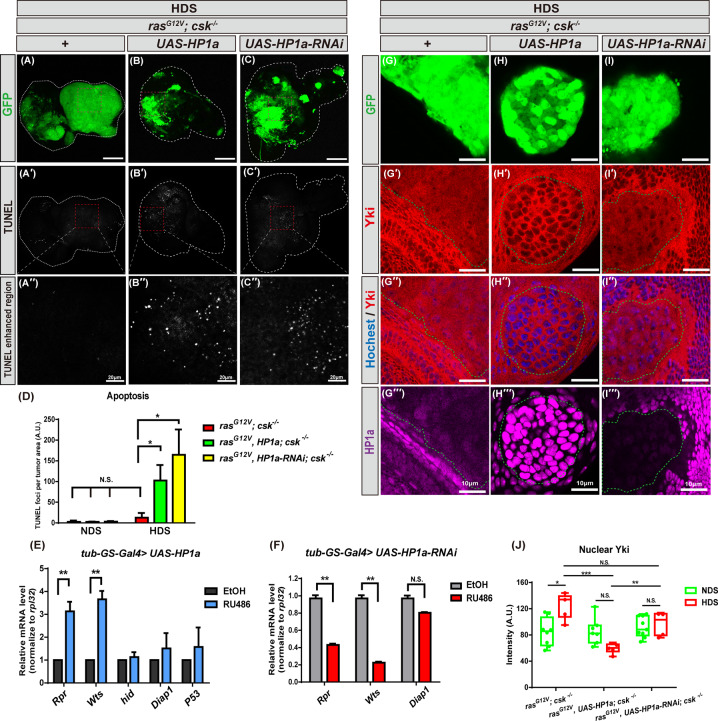
Increased heterochromatin formation increases apoptosis in tumor cells under HDS. **A–D** Third instar eye discs of *ras*^*G12V*^*; csk*^*−/−*^ and *ras*^*G12V*^*, HP1a; csk*^*−/−*^ and *ras*^*G12V*^*, HP1a-RNAi; csk*^*−/−*^ flies fed HDS with GFP-labeled tumor cells (green) and TUNEL staining (gray). Scale bar: 100 μm unless specified otherwise. **A**–**A**′′ *ras*^*G12V*^; *csk*^*−/−*^. **B**–**B**′′ *ras*^*G12V*^*, HP1a*; *csk*^*−/−*^. **C**–**C**′′ *ras*^*G12V*^*, HP1a-RNAi*; *csk*^*−/−*^. **D** Quantification of fluorescent TUNEL staining, which indicates apoptosis, in tumor cells from *ras*^*G12V*^*; csk*^*−/−*^ and *ras*^*G12V*^*, HP1a; csk*^*−/−*^ and *ras*^*G12V*^*, HP1a-RNAi; csk*^*−/−*^ flies fed HDS. All results analyzed and presented reflect data from three independent experiments (*n* = 15). **E**, **F** Relative mRNA expression of flies carrying the *tub-GS-Gal4* driver outcrossed with *UAS-HP1a* or *UAS-HP1a-RNAi* flies that were fed RU486 for 4 days. Control flies received food with EtOH. Total RNA was isolated from 30 adult fly heads of the same gender after 4 days of RU486 treatment. Multiple *t*-tests were used to derive *p* values. Results are shown as mean ± SD of individual eye discs or heads. **G–J** Third instar eye discs of *ras*^*G12V*^*; csk*^*−/−*^ and *ras*^*G12V*^*, HP1a; csk*^*−/−*^ and *ras*^*G12V*^*, HP1a-RNAi; csk*^*−/−*^ flies fed HDS with GFP-labeled tumor cells (green), Yki (red), and Hochest staining (blue). Scale bar: 10 μm. **G**–**G**′′′ *ras*^*G12V*^; *csk*^*−/−*^. **H**–**H**′′′ *ras*^*G12V*^*, HP1a*; *csk*^*−/−*^. **I**–**I**′′′ *ras*^*G12V*^*, HP1a-RNAi*; *csk*^*−/−*^. **J** Quantification of fluorescent nuclear Yki staining, from *ras*^*G12V*^*; csk*^*−/−*^ and *ras*^*G12V*^*, HP1a; csk*^*−/−*^ and *ras*^*G12V*^*, HP1a-RNAi; csk*^*−/−*^ flies fed NDS (see Supplement Fig. 4D–F′′′) or HDS. All results analyzed and presented reflect data from three independent experiments (*n* = 15). Asterisks indicate statistically significant differences via two-way ANOVA with relevant paired controls (*p < 0.05; **p < 0.01; ***p < 0.001). GFP green fluorescent protein, HDS high dietary sugar, NDS normal dietary sugar, NS not significant.

We next investigated whether HP1a-mediated heterochromatin formation suppressed HDS-induced tumor growth by regulating apoptosis. Using the terminal deoxynucleotidyl transferase dUTP nick end labeling (TUNEL) assay for detecting DNA fragmentation by labeling the 3′-hydroxyl termini in double-stranded DNA at the final stage of programmed cell death, we found that apoptosis was reduced in tumor cells under HDS (Fig. 5A–A′′), which is consistent with previous findings [23]. Importantly, increasing heterochromatin formation by HP1a overexpression in tumor-bearing flies significantly increased apoptosis specifically in the HDS condition (Supplement Fig. 4A–B′′). Moreover, drastic heterochromatin reduction also led to apoptosis in tumor cells under HDS (Fig. 5C–C′′, D). To further dissect the molecular mechanism by which HP1a-mediated heterochromatin formation downregulates apoptosis, we examined whether heterochromatin regulates apoptosis-related genes using an RU486-inducible HP1a overexpression or knockdown system in normal adult flies. We first verified that HP1a mRNA levels were drastically increased or decreased in flies with inducible HP1a or HP1a-RNAi, respectively, when treated with RU486. Moreover, *Reaper* (*Rpr*) and *Warts* (*Wts)*, two key pro-apoptotic genes, were increased in HP1a-overexpressing flies, but decreased in HP1a knockdown flies (Fig. 5E, F), suggesting that HP1a-mediated heterochromatin formation upregulates apoptotic pathway genes.

Wts is a key regulator that controls the nuclear localization of transcriptional coactivator Yorkie (Yki) in the Hippo pathway, which drives tumor growth [28]. Therefore, we next determined the activity of Hippo signaling by Yki immunohistochemistry in Ras/Src tumor cells. We found that Yki nuclear localization was increased in Ras/Src tumor clones from animals fed HDS compared to those fed NDS (Supplement Fig. 4). Interestingly, under conditions of NDS, HP1a overexpression or knockdown did not alter Yki nuclear localization in tumor cells, compared to control Ras/Src tumor cells. Importantly, in flies fed HDS, Yki nuclear localization was reduced in Ras/Src tumor clones overexpressing HP1a or HP1a-RNAi, compared to control Ras/Src tumor cells (Fig. 5G–I′′′). Moreover, under conditions of HDS, HP1a overexpression reduced more Yki nuclear accumulation than HP1a knockdown (Fig. 5J), suggesting that HP1a-mediated heterochromatin formation suppresses HDS-induced tumor growth by downregulating Yki nuclear localization in Hippo signaling.

Taken together, these results suggest that increased heterochromatin formation suppresses HDS-induced tumor growth by inducing apoptosis through downregulation of Hippo signaling and upregulation of apoptosis-related genes.

### Increased heterochromatin formation reduces HDS-induced wingless signaling in tumor cells

**Fig. 6:**
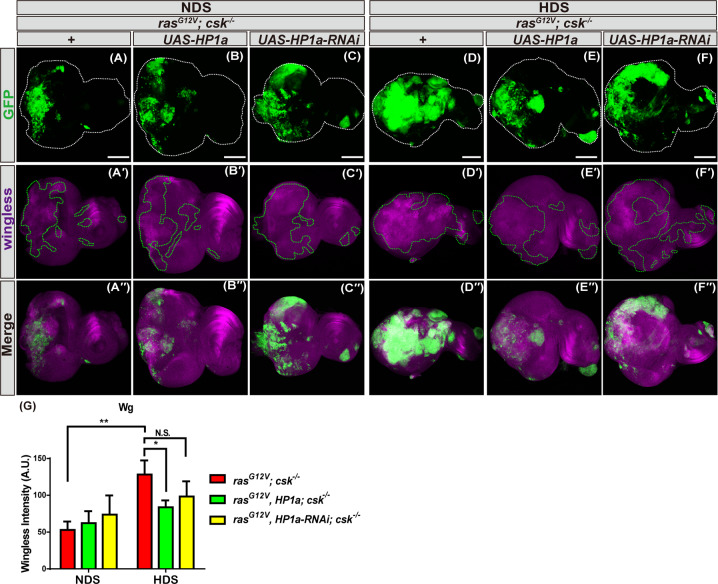
Increased heterochromatin formation inhibits HDS-induced wingless expression in tumor cells. Third instar eye discs of *ras*^*G12V*^*; csk*^*−/−*^ and *ras*^*G12V*^*, HP1a; csk*^*−/−*^ and *ras*^*G12V*^*, HP1a-RNAi; csk*^*−/−*^ flies fed NDS or HDS with GFP-labeled tumor cells (green) and wingless staining (magenta). Scale bar: 100 μm. **A–A′′, D–D′′** *ras*^*G12V*^*; csk*^*−/−*^. **B–B′′, E–E′′** *ras*^*G12V*^*, HP1a*; *csk*^*−/−*^. **C–C′′, F–F′′** *ras*^*G12V*^*, HP1a-RNAi*; *csk*^*−/−*^. **G** Quantification of wingless immunofluorescent signal in tumor cells of *ras*^*G12V*^*; csk*^*−/−*^ and *ras*^*G12V*^*, HP1a; csk*^*−/−*^ and *ras*^*G12V*^*, HP1a-RNAi; csk*^*−/−*^ flies fed NDS or HDS. All results analyzed and presented reflect data from five independent experiments (*n* = 25). Results are shown as mean ± SD of individual eye discs. Asterisks indicate statistically significant differences via two-way ANOVA with relevant paired controls (*p < 0.05; **p < 0.01). GFP green fluorescent protein, HDS high dietary sugar, NDS normal dietary sugar, NS not significant.

It has been shown that wingless signaling is important for HDS-induced tumor growth in *Drosophila* [23]. To determine whether HP1a-mediated heterochromatin formation suppresses tumor growth by regulating wingless, we examined wingless expression in tumor tissues from flies with increased or decreased HP1a-mediated heterochromatin levels. Consistent with previous findings [23], wingless was highly upregulated in Ras/Src tumor clones from animals fed HDS compared to those fed NDS. Importantly, in flies fed HDS, wingless expression was reduced in Ras/Src tumor clones overexpressing HP1a, but not in those overexpressing HP1a-RNAi, compared to control Ras/Src tumor cells. Moreover, tumor clones with increased or decreased heterochromatin levels, from flies fed NDS, did not show significant changes in wingless expression (Fig. 6), indicating that wingless signaling may play an important role in tumor suppression under conditions of HDS, rather than in the context of NDS. Overall, these results suggest that increased heterochromatin formation in tumor cells suppresses HDS-induced wingless signaling and thereby inhibits tumor progression.

### Increased heterochromatin formation reduces HDS-induced genome instability in tumor cells

**Fig. 7:**
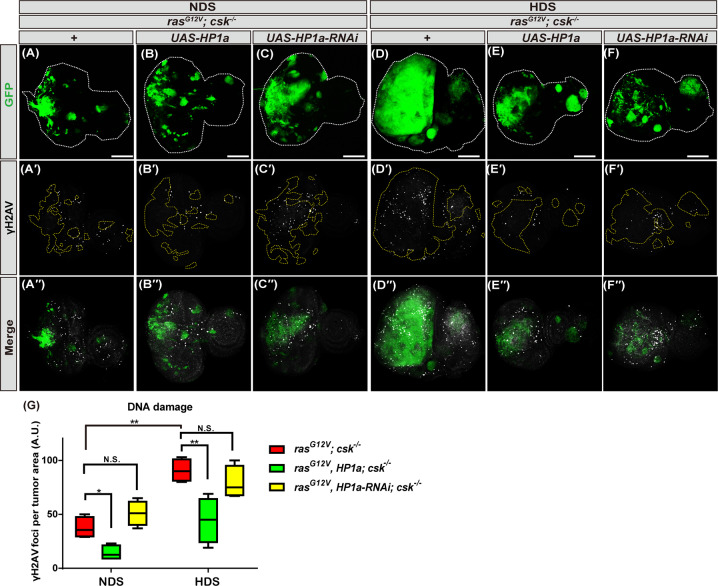
Increased heterochromatin formation reduces HDS-induced DNA damage in tumor cells. Third instar eye discs of *ras*^*G12V*^*; csk*^*−/−*^ and *ras*^*G12V*^*, HP1a; csk*^*−/−*^ and *ras*^*G12V*^*, HP1a-RNAi; csk*^*−/−*^ flies fed NDS or HDS with GFP-labeled tumor cells (green) and γH2AV staining (gray). Scale bar: 100 μm. **A–A′′, D–D′′** *ras*^*G12V*^*; csk*^*−/−*^. **B–B′′, E–E′′** *ras*^*G12V*^*, HP1a*; *csk*^*−/−*^. **C–C′′, F–F′′** *ras*^*G12V*^*, HP1a-RNAi*; *csk*^*−/−*^. **G** Quantification of γH2AV immunofluorescent signal indicating DNA damage in tumor cells of *ras*^*G12V*^*; csk*^*−/−*^ and *ras*^*G12V*^*, HP1a; csk*^*−/−*^ and *ras*^*G12V*^*, HP1a-RNAi; csk*^*−/−*^ flies fed NDS or HDS. All results analyzed and presented reflect data from three independent experiments (*n* = 15). Results are shown as mean ± SD of individual eye discs. Asterisks indicate statistically significant differences via two-way ANOVA with relevant paired controls (*p < 0.05; **p < 0.01). GFP green fluorescent protein, HDS high dietary sugar, NDS normal dietary sugar, NS not significant.

Genome instability plays an important role in promoting tumorigenesis [29]. We next investigated whether genome instability is increased in tumor cells under HDS, and whether heterochromatin formation suppresses tumor progression through improving genome stability in Ras/Src tumor cells under HDS. Indeed, the number of foci of γ-H2Av, a DNA damage marker for double-stranded DNA breaks, was increased with HDS compared to NDS. Moreover, γ-H2Av foci were dramatically decreased with HP1a overexpression in Ras/Src tumor cells from flies fed either NDS or HDS. On the other hand, the number of γ-H2Av foci did not change significantly in Ras/Src tumor cells with HP1a knockdown as compared to control Ras/Src tumor cells from flies fed either NDS or HDS (Fig. 7). These results suggest that, under conditions of HDS, heterochromatin formation maintains or restores genome stability and promotes tumor suppression.

## Discussion

**Fig. 8:**
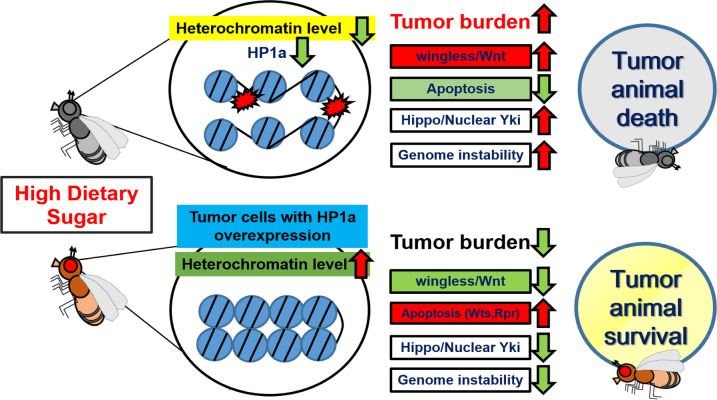
Heterochromatin formation suppresses HDS-induced tumor progression by downregulating wingless/Wnt and Hippo signaling, increasing apoptosis, and maintaining genome stability. Under conditions of HDS, increasing HP1a-mediated heterochromatin formation in tumor cells can decrease tumor burden and increase survival of tumor-bearing flies. Moreover, HDS reduces heterochromatin formation in tumor cells. Mechanistically, heterochromatin formation suppresses HDS-induced tumor progression by decreasing wingless expression and nuclear Yki accumulation, inducing apoptosis and maintaining genome stability.

There are over 650 million clinically obese adults worldwide, and over-consumption of added sugars in the diet plays a major role in obesity [20]. Moreover, HDS-induced type II diabetes mellitus (T2D) and metabolic disorders play critical roles in cancer development and progression. However, the epigenetic mechanisms controlling HDS-induced tumor progression are complex and not fully understood. Using genetically tractable *Drosophila* tumor systems, we found that heterochromatin formation suppresses HDS-aggravated tumor growth. Increasing HP1a-mediated heterochromatin formation suppresses developmental delay and lethality in HDS-fed Ras/Src and Ras/scrib tumor-bearing flies; this occurs not only by increasing tumor apoptosis but also by reducing wingless and Hippo signaling, as well as maintaining genome stability (Fig. 8). Our research identified epigenetic regulatory mechanisms involving HP1a-mediated heterochromatin in cancer development. The structure and function of HP1a are highly conserved from *Drosophila* to humans, and HP1a possesses important and evolutionarily conserved functions across species [30]. HP1a is downregulated in several human cancers, including breast cancer [31], leukemia [32], and brain cancer [33]. Consistently, we found that HDS reduces heterochromatin formation to promote tumor progression, and that restored heterochromatin levels can suppress HDS-induced tumor growth, developmental delay, and lethality. However, additional studies testing more pro-heterochromatin factors such as Su(var)3–9 are required to confirm the role of heterochromatin formation in HDS-induced tumor progression.

In this study, we have demonstrated extensively the in vivo functions of HP1a, the evolutionarily conserved key molecule in heterochromatin formation, with four different *HP1a* genetic manipulation, in mediating HDS-induced tumorigenesis. Indeed, we found that both increased and decreased heterochromatin formation, via HP1a overexpression by expressing *UAS-HP1a* (120% increase)/*hs-HP1a* (20% increase) and *UAS-HP1a-RNAi* knockdown (65% decrease) in tumor cells, respectively, can suppress HDS-dependent tumorigenesis. However, we think that different underlying mechanisms exist for the suppression of HDS-dependent tumorigenesis between HP1a overexpression and knockdown, and three possible explanations are as follows. Firstly, HP1a protein levels were decreased by 65% in HP1a knockdown tumor clones under NDS (Fig. 1), which led to suppression of HDS-induced tumorigenesis. On the other hand, HP1a protein levels were reduced by 50% in Ras/Src tumor cells under HDS compared to NDS (Fig. 2), which resulted in promotion of HDS-induced tumorigenesis. Consistently, reducing HP1 levels by half (50% decrease) as in *ras*^*G12V*^*, HP1a*^*+/−*^*; csk*^*−/−*^ also did not suppress HDS-induced tumor progression (Supplement Fig. 3). Therefore, it is likely that such a 15% difference in HP1a levels may contribute to different outcomes of HDS-dependent tumorigenesis. Additional studies will be required to determine the switch between suppressing and promoting HDS-dependent tumorigenesis with regards to reduced HP1a levels in other cancer model systems and human cancers. Secondly, knocking down HP1a also led to significant increase of apoptosis, as did overexpression of HP1a (Fig. 5). However, the underlying pro-apoptotic mechanism might be different, as the levels of *Rpr* and *Wts* mRNA were increased following HP1a overexpression, but reduced following HP1a knockdown, in adult flies (Fig. 5), suggesting that different mechanisms were at play even through similar outcome, i.e. increase of apoptosis, was observed. Moreover, we found that under HDS, increased heterochromatin formation via HP1a overexpression led to decreased nuclear localization of Yki in Hippo signaling, but RNAi against HP1a did not have any effect on the expression level or distribution of Yki (Fig. 5), further suggesting that HP1a overexpression and knockdown may employ different apoptotic mechanisms in suppressing HDS-induced tumorigenesis. Previous research in a breast cancer model demonstrated that bi-phasic expression of HP1a during breast cancer progression plays a role in tumorigenesis [34]. Moreover, depletion of HP1a causes apoptosis [11]. This may hold the explanation as to why the two seemly contradicting manipulations, i.e. overexpression vs. knocking down HP1a, led to similar outcomes in terms of suppressing HDS-dependent tumorigenesis. In other words, decreasing heterochromatin formation by HP1a knockdown in tumor cells may increase apoptosis in tumor cells and decrease the HDS-induced tumor burden in the Ras/Src tumor system. Thirdly, HP1a overexpression did reduce more wingless expression and DNA damage than HP1a knockdown (Figs. 5–7). More studies are needed to further explore the role of heterochromatin formation in cancers associated with HDS-induced metabolic disorders.

Interestingly, Ras/Src tumor clones can be observed in the adult eyes, (Supplement Fig. 5), suggesting that not all of the tumor cells are completely eliminated by apoptosis. Moreover, previous study has found that HDS blocks apoptosis in Ras/Src tumor [23] (Supplement Fig. 6). However, functions of apoptosis on HDS-induced tumor progression remain unclear. Understanding heterochromatin-mediated apoptosis on HDS-induced tumor progression will require more studies. Additional studies will be required to confirm the effect of HP1 overexpression or downregulation on clone size and eclosion rate of tumor-bearing animals, if tumor cells are prevented from dying, e.g., by coexpression of p35.

In this study, we showed that increased heterochromatin formation in Ras/Src tumor cells suppresses HDS-induced tumor growth (Fig. 3), reduces proliferation under NDS (Fig. 4), and increases apoptosis under HDS (Fig. 5). However, tumor clones with increased heterochromatin do not show autonomous effect on cell proliferation and apoptotic cell death, as accessed by PH3 and TUNEL staining, respectively (Figs. 4 and 5). Mechanisms underlying such nonautonomous effect in Ras/Src tumor system are not clear. Apoptotic cells can produce nonautonomous signals, including wingless, dpp, and TNF, to induce compensatory proliferation and apoptosis of the neighboring cells in *Drosophila* wing imaginal disc [35–37]. Thus, heterochromatin levels in tumor cells may also regulate global changes in proliferation or apoptosis in the tumor containing imaginal eye discs. Interestingly, we found that wingless signaling is upregulated by HDS, and increased heterochromatin formation reduces HDS-induced wingless signaling in tumor cells (Fig. 6), suggesting that wingless signaling may be involved to coordinate global changes of proliferation or apoptosis in the tumor containing eye discs. Future studies will be necessary to understand the mechanisms underlying heterochromatin-mediated nonautonomous proliferation and apoptosis effects on HDS-induced tumor progression.

Previous study has shown that expression of wg-RNAi or a dominant-negative form of TCF suppressed HDS-induced Ras/Src tumor growth, suggesting that canonical wg signaling is required for HDS-induced tumor growth [23]. Understanding heterochromatin-mediated wg signaling on HDS-induced tumor progression will require additional studies. It is necessary to confirm the effect of heterochromatin formation on HDS-tumor progression by coexpression of wg-RNAi or dominant-negative TCF.

HP1a-mediated heterochromatin protects genome stability [38]. Moreover, genome instability is a driver of tumor progression [10]. Consistently, we found that maintenance of genome stability by heterochromatin formation plays an important role in suppression of HDS-induced tumor progression.

Diet is an important environmental cue that may impact epigenomics. Evidence has indicated that transient hyperglycemia regulates expression of certain genes by altering chromatin states [39], and our study showed that HDS decreases heterochromatin formation in *Drosophila*. Interestingly, we found that HDS decreases heterochromatin formation in wild-type animals as well as in tumor cells under HDS. Together, the results of our studies and the work of others implicate HDS-induced epigenetic changes in promotion of tumor progression through upregulation of oncogenic genes or downregulation of tumor suppressor genes.

Overall, our study highlights heterochromatin formation as an important tumor suppressor that can inhibit HDS-induced tumor progression. We revealed that increased heterochromatin levels suppress tumor progression likely through modulation of wingless and Hippo signaling, apoptosis, and genomic stability. We also demonstrated epigenetic mechanisms by which HP1a-mediated heterochromatin suppresses HDS-induced tumor progression. Epigenetic cancer therapies, such as DNA methyltransferase (DNMT) inhibitors, have been approved by the FDA for the treatment of various cancers. Thus, our studies support the potential of heterochromatin formation as a promising epigenetic therapeutic target for the treatment of cancers associated with HDS-induced metabolic disorders.

## Supplementary information

Supplementary Figure Legends

Supplement Figure 1

Supplement Figure 2

Supplement Figure 3

Supplement Figure 4

Supplement Figure 5

Supplement Figure 6

## Data Availability

All data related to this study are available from the corresponding author upon request.

## References

[1] Bray F, Ferlay J, Soerjomataram I, Siegel RL, Torre LA, Jemal A (2018). Global cancer statistics 2018: GLOBOCAN estimates of incidence and mortality worldwide for 36 cancers in 185 countries. CA Cancer J Clin.

[2] Patterson CC, Karuranga S, Salpea P, Saeedi P, Dahlquist G, Soltesz G (2019). Worldwide estimates of incidence, prevalence and mortality of type 1 diabetes in children and adolescents: results from the International Diabetes Federation Diabetes Atlas, 9th edition. Diabetes Res Clin Pract.

[3] Sumiyoshi M, Sakanaka M, Kimura Y (2006). Chronic intake of high-fat and high-sucrose diets differentially affects glucose intolerance in mice. J Nutr.

[4] Hanahan D, Weinberg RA (2000). The hallmarks of cancer. Cell.

[5] Warburg O (1956). On respiratory impairment in cancer cells. Science.

[6] Vander Heiden MG, Cantley LC, Thompson CB (2009). Understanding the Warburg effect: the metabolic requirements of cell proliferation. Science.

[7] Hirabayashi S, Cagan RL (2015). Salt-inducible kinases mediate nutrient-sensing to link dietary sugar and tumorigenesis in Drosophila. eLife.

[8] Mullen AR, Wheaton WW, Jin ES, Chen PH, Sullivan LB, Cheng T (2011). Reductive carboxylation supports growth in tumour cells with defective mitochondria. Nature.

[9] Zhang J, Nuebel E, Daley GQ, Koehler CM, Teitell MA (2012). Metabolic regulation in pluripotent stem cells during reprogramming and self-renewal. Cell Stem Cell.

[10] Hu CM, Tien SC, Hsieh PK, Jeng YM, Chang MC, Chang YT (2019). High glucose triggers nucleotide imbalance through O-GlcNAcylation of key enzymes and induces kras mutation in pancreatic cells. Cell Metab.

[11] De Lucia F, Ni JQ, Vaillant C, Sun FL (2005). HP1 modulates the transcription of cell-cycle regulators in Drosophila melanogaster. Nucleic Acids Res.

[12] Hahn SA, Schutte M, Hoque AT, Moskaluk CA, da Costa LT, Rozenblum E (1996). DPC4, a candidate tumor suppressor gene at human chromosome 18q21.1. Science.

[13] Feinberg AP (2007). Phenotypic plasticity and the epigenetics of human disease. Nature.

[14] Kirschmann DA, Lininger RA, Gardner LM, Seftor EA, Odero VA, Ainsztein AM (2000). Down-regulation of HP1Hsalpha expression is associated with the metastatic phenotype in breast cancer. Cancer Res.

[15] Dialynas GK, Vitalini MW, Wallrath LL (2008). Linking Heterochromatin Protein 1 (HP1) to cancer progression. Mutat Res.

[16] Goodsell DS (1999). The molecular perspective: the ras oncogene. Oncologist.

[17] Vidal M, Cagan RL (2006). Drosophila models for cancer research. Curr Opin Genet Dev.

[18] Pagliarini RA, Xu T (2003). A genetic screen in Drosophila for metastatic behavior. Science.

[19] Adams MD, Celniker SE, Holt RA, Evans CA, Gocayne JD, Amanatides PG (2000). The genome sequence of Drosophila melanogaster. Science.

[20] Gonzalez C (2013). Drosophila melanogaster: a model and a tool to investigate malignancy and identify new therapeutics. Nat Rev Cancer.

[21] Musselman LP, Fink JL, Narzinski K, Ramachandran PV, Hathiramani SS, Cagan RL (2011). A high-sugar diet produces obesity and insulin resistance in wild-type Drosophila. Dis Model Mech.

[22] Hsu TH, Yang CY, Yeh TH, Huang YC, Wang TW, Yu JY (2017). The Hippo pathway acts downstream of the Hedgehog signaling to regulate follicle stem cell maintenance in the Drosophila ovary. Sci Rep.

[23] Hirabayashi S, Baranski TJ, Cagan RL (2013). Transformed Drosophila cells evade diet-mediated insulin resistance through wingless signaling. Cell.

[24] Wu M, Pastor-Pareja JC, Xu T (2010). Interaction between Ras(V12) and scribbled clones induces tumour growth and invasion. Nature.

[25] Larson K, Yan SJ, Tsurumi A, Liu J, Zhou J, Gaur K (2012). Heterochromatin formation promotes longevity and represses ribosomal RNA synthesis. PLoS Genet.

[26] Eissenberg JC, Morris GD, Reuter G, Hartnett T (1992). The heterochromatin-associated protein HP-1 is an essential protein in Drosophila with dosage-dependent effects on position-effect variegation. Genetics.

[27] Kim JY, Jeong HS, Chung T, Kim M, Lee JH, Jung WH (2017). The value of phosphohistone H3 as a proliferation marker for evaluating invasive breast cancers: a comparative study with Ki67. Oncotarget.

[28] Zheng Y, Pan D (2019). The Hippo signaling pathway in development and disease. Dev Cell.

[29] Palazzo L, Ahel I (2018). PARPs in genome stability and signal transduction: implications for cancer therapy. Biochem Soc Trans.

[30] Norwood LE, Grade SK, Cryderman DE, Hines KA, Furiasse N, Toro R (2004). Conserved properties of HP1(Hsalpha). Gene.

[31] Hendrix MJ, Seftor EA, Kirschmann DA, Seftor RE (2000). Molecular biology of breast cancer metastasis. Molecular expression of vascular markers by aggressive breast cancer cells. Breast Cancer Res.

[32] Popova EY, Claxton DF, Lukasova E, Bird PI, Grigoryev SA (2006). Epigenetic heterochromatin markers distinguish terminally differentiated leukocytes from incompletely differentiated leukemia cells in human blood. Exp Hematol.

[33] Pomeroy SL, Tamayo P, Gaasenbeek M, Sturla LM, Angelo M, McLaughlin ME (2002). Prediction of central nervous system embryonal tumour outcome based on gene expression. Nature.

[34] Lee YH, Ann DK (2015). Bi-phasic expression of Heterochromatin Protein 1 (HP1) during breast cancer progression: Potential roles of HP1 and chromatin structure in tumorigenesis. J Nat Sci.

[35] Takino K, Ohsawa S, Igaki T (2014). Loss of Rab5 drives non-autonomous cell proliferation through TNF and Ras signaling in Drosophila. Dev Biol.

[36] Ryoo HD, Gorenc T, Steller H (2004). Apoptotic cells can induce compensatory cell proliferation through the JNK and the Wingless signaling pathways. Dev Cell.

[37] La Marca JE, Richardson HE (2020). Two-faced: roles of JNK signalling during tumourigenesis in the Drosophila model. Front Cell Dev Biol.

[38] Yan S-J, Lim SJ, Shi S, Dutta P, Li WX (2011). Unphosphorylated STAT and heterochromatin protect genome stability. FASEB J.

[39] Ling C, Groop L (2009). Epigenetics: a molecular link between environmental factors and type 2 diabetes. Diabetes.

