# Supplementary material for: HP1a-mediated heterochromatin formation inhibits high dietary sugar-induced tumor progression
Source: Cell Death Dis. 2021 Dec 6;12(12):1130. doi: 10.1038/s41419-021-04414-z (PMC8645608; doi:10.1038/s41419-021-04414-z)
Supplement: Supplementary file 1 — Supplementary Figure Legends [file 41419_2021_4414_MOESM1_ESM.docx]

**Supplement Fig. 1: Increased HP1a-mediated heterochromatin formation decreases HDS-induced developmental delay and increases tumor bearing flies survival in the Ras/scrib tumor model**

**(A-D)** Pupation rate of animals fed NDS and HDS, respectively, with the following genotypes: (A) *lacZ*, (B) *ras^G12V^; scrib^-/-^*, (C) *ras^G12V^, HP1a; scrib^-/-^*, (D) *ras^G12V^, HP1a-RNAi; scrib^-/-^*. Each genotype contains at least 150 flies.

Third instar eye discs of (E-H) *ras^G12V^, HP1a; scrib^-/-^* flies and (I-L) *ras^G12V^, HP1a-RNAi; scrib^-/-^* flies fed NDS with GFP-labeled tumor cells (green) and HP1a (magenta) and H3K9me2 (red) immunostaining. Scale bar: 10 μm. GFP, green fluorescent protein; HDS, high dietary sugar; NDS, normal dietary sugar.

**Supplement Fig. 2: The UAS-HP1a-RNAi line (VDRC 31995) driven by ey-Gal4 decreases the HP1a levels in the eye discs**

Third instar eye discs of flies carrying the *ey-Gal4* driver outcrossed with (A-D) *UAS-lacZ,* (E-F) *UAS-HP1a-RNAi* (VDRC 31995), (G-H) *UAS-HP1a-RNAi* (BDSC 33400), (I-J) *UAS-HP1a-RNAi* (BDSC 36792), with ß-galactosidase (green), HP1a (red) and Hochest staining (blue). Scale bar: 100μm.

**Supplement Fig. 3: Increased HP1a-mediated heterochromatin formation decreases HDS-induced tumor growth and lethality of Ras/Src tumor bearing flies**

**(A-C)** Eclosion rate of tumor-bearing animals, fed NDS or HDS, with the following genotypes: (A) *ras^G12V^; csk^-/-^*, (B) *ras^G12V^, hs-HP1a; csk^-/-^*, (C) *ras^G12V^, HP1a^+/-^; csk^-/-^* (Red line, female; Blue line: male). Each genotype contains at least 150 flies.

**(D)** The survival rates of animals, fed NDS or HDS, with the following genotypes: *lacZ,* and *ras^G12V^; csk^-/-^* and *ras^G12V^*, *hs-HP1a; csk^-/-^*, and *ras^G12V^, HP1a^+/-^; csk^-/-^*. All results analyzed and presented reflect data from four independent experiments. Each genotype contains at least 150 flies.

**(E-J)** Third instar eye discs of *ras^G12V^; csk^-/-^* and *ras^G12V^, hs-HP1a; csk^-/-^* and *ras^G12V^, HP1a^+/-^; csk^-/-^* flies fed NDS or HDS; tumor cells are labeled with GFP (green). Scale bar: 100 μm.

**(K-L)** Percentage of GFP-positive tumor cells normalized to total eye disc area from *ras^G12V^; csk^-/-^* and *ras^G12V^, hs-HP1a; csk^-/-^* and *ras^G12V^, HP1a^+/-^; csk^-/-^* female or male flies fed NDS or HDS. All results analyzed and presented reflect data from three independent experiments (n=15).

Results are shown as mean ± SD. Asterisks indicate statistically significant differences (*, p < 0.05; **, p < 0.01; ***, p < 0.001). GFP, green fluorescent protein; HDS, high dietary sugar; NDS, normal dietary sugar. N.S., not significant.

**Supplement Fig. 4: Heterochromatin formation does not alter apoptosis and nuclear Yki in tumor cells from flies fed NDS**

**(A-C′′)** Third instar eye discs of *ras^G12V^; csk^-/-^* and *ras^G12V^, HP1a; csk^-/-^* and *ras^G12V^, HP1a-RNAi; csk^-/-^* flies fed NDS with GFP-labeled tumor cells (green) and TUNEL staining (gray). Scale bar: 100 μm unless specified otherwise. (A-A′′) *ras^G12V^*; *csk^-/-^*. (B-B′′) *ras^G12V^, HP1a*; *csk^-/-^*. (C-C′′) *ras^G12V^, HP1a-RNAi*; *csk^-/-^*.

**(D-F′′′)** Third instar eye discs of *ras^G12V^; csk^-/-^* and *ras^G12V^, HP1a; csk^-/-^* and *ras^G12V^, HP1a-RNAi; csk^-/-^* flies fed HDS with GFP-labeled tumor cells (green), Yki (red) and Hochest staining (blue). Scale bar: 10 μm. (D-D′′′) *ras^G12V^*; *csk^-/-^*. (E-E′′′) *ras^G12V^, HP1a*; *csk^-/-^*. (F-F′′′) *ras^G12V^, HP1a-RNAi*; *csk^-/-^*.

GFP, green fluorescent protein; NDS, normal dietary sugar.

**Supplement Fig. 5: Ras/Src tumor cells can survive after eclosion**

Eyes of *ras^G12V^; csk^-/-^* and *ras^G12V^, HP1a; csk^-/-^* and *ras^G12V^, HP1a-RNAi; csk^-/-^* female adult flies fed NDS or HDS with GFP-labeled tumor cells (green). (A-A′, D-D′) *ras^G12V^*; *csk^-/-^*. (B-B′, E-E′) *ras^G12V^, HP1a*; *csk^-/-^*. (C-C′, F-F′) *ras^G12V^, HP1a-RNAi*; *csk^-/-^*. GFP, green fluorescent protein; HDS, high dietary sugar; NDS, normal dietary sugar.

**Supplement Fig. 6: Increased heterochromatin formation upregulates the apoptotic pathway and reduces Yki nuclear localization to promote apoptosis in tumor cells under HDS**

Model: Increasing heterochromatin formation suppresses HDS-induced tumor progression likely by upregulating the apoptosis-related genes and reducing Yki nuclear localization to promote apoptosis in tumor cells under HDS.
