# Supplementary figures and images for: HP1a-mediated heterochromatin formation inhibits high dietary sugar-induced tumor progression
Source: Cell Death Dis. 2021 Dec 6;12(12):1130. doi: 10.1038/s41419-021-04414-z (PMC8645608; doi:10.1038/s41419-021-04414-z)

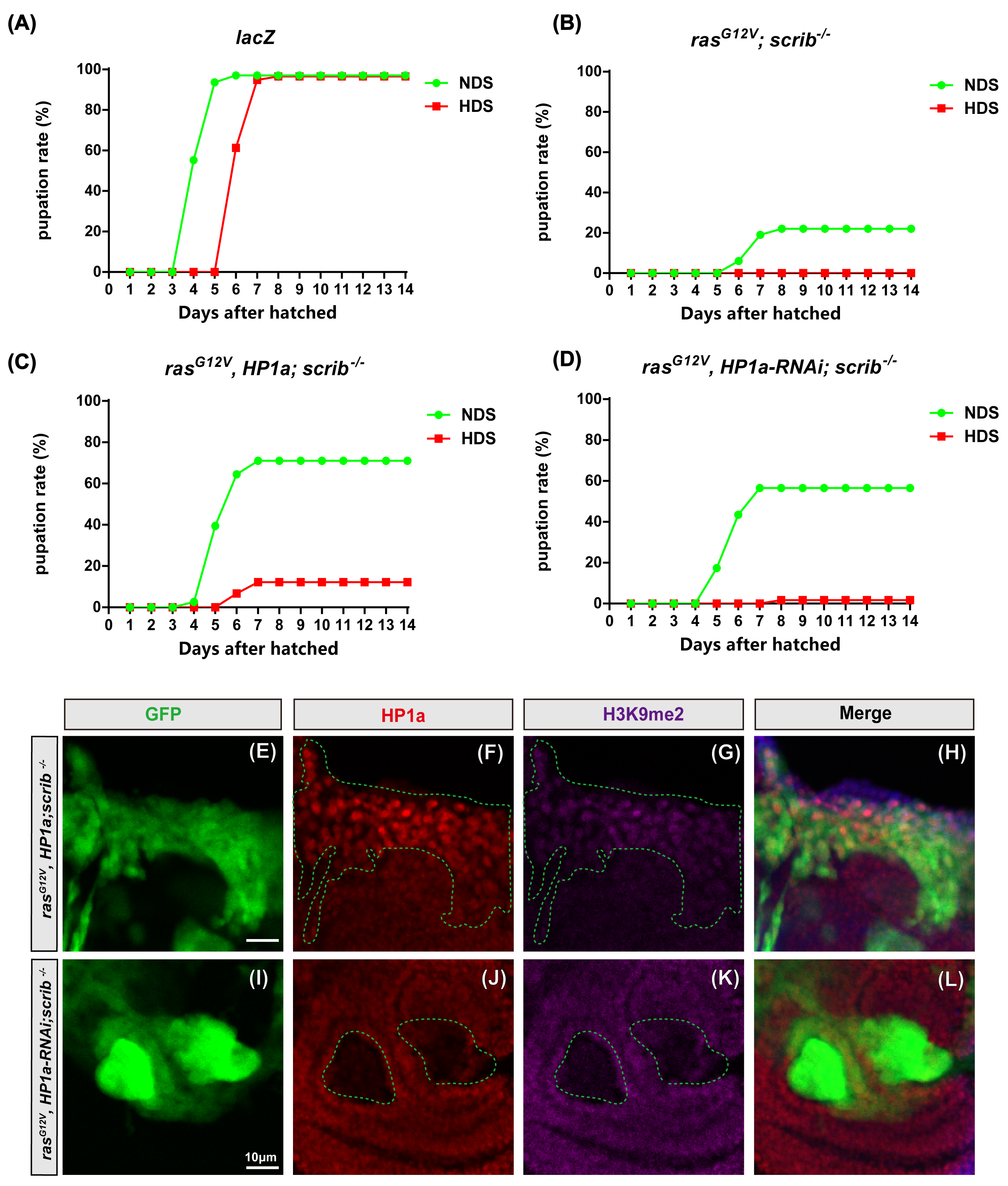

Supplement: Supplementary file 2 — Supplement Figure 1 [file 41419_2021_4414_MOESM2_ESM.tif]

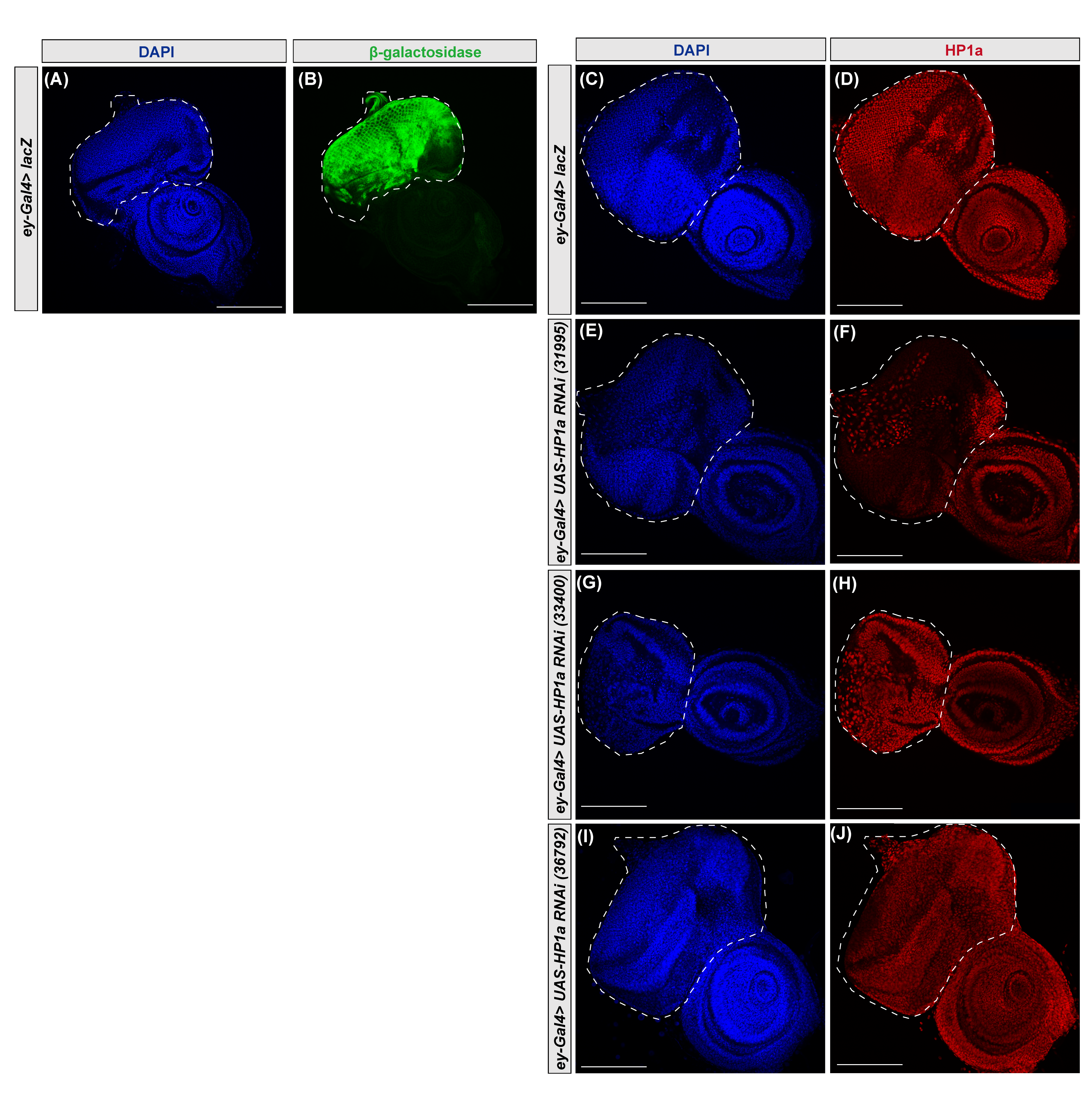

Supplement: Supplementary file 3 — Supplement Figure 2 [file 41419_2021_4414_MOESM3_ESM.tif]

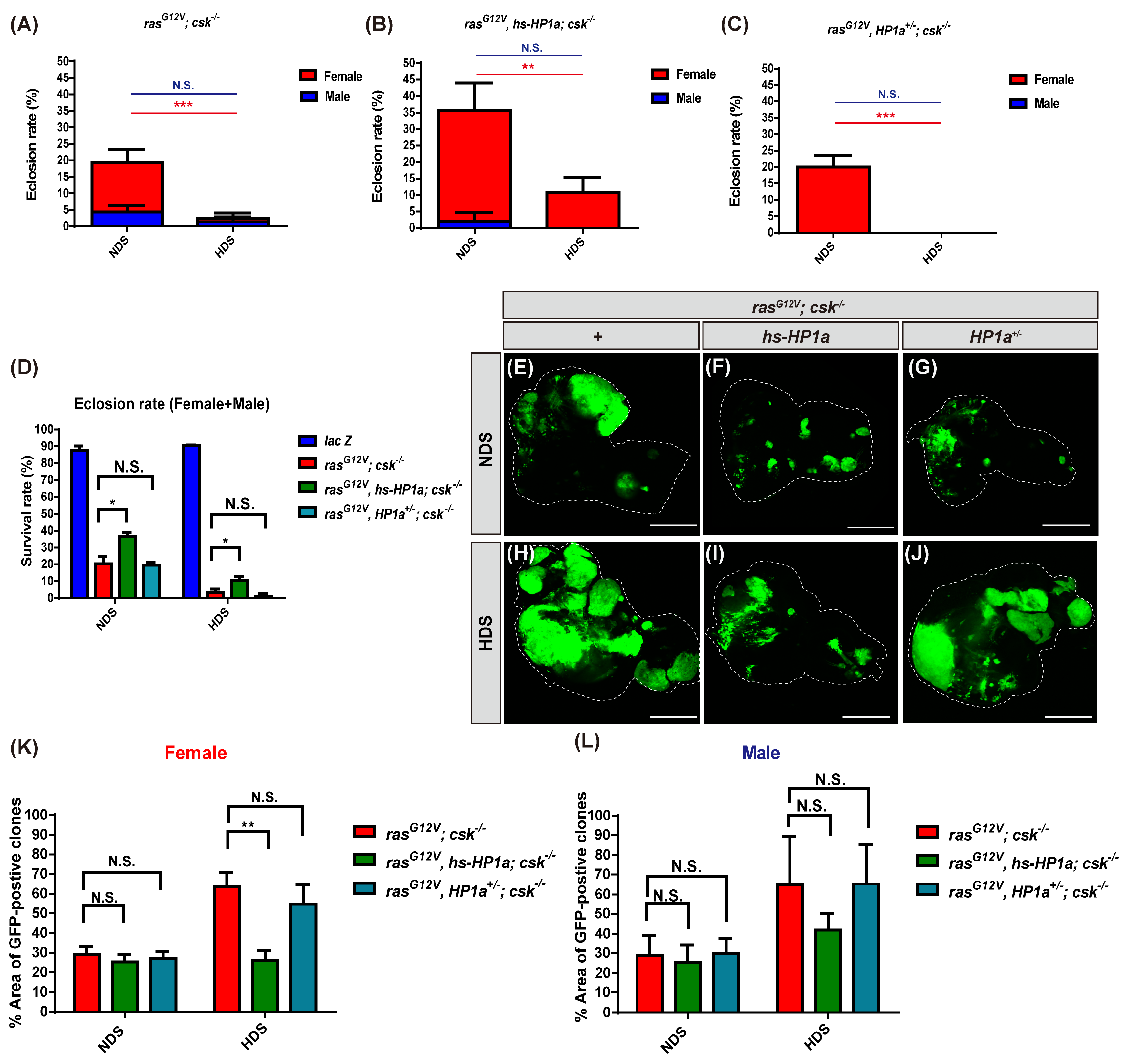

Supplement: Supplementary file 4 — Supplement Figure 3 [file 41419_2021_4414_MOESM4_ESM.tif]

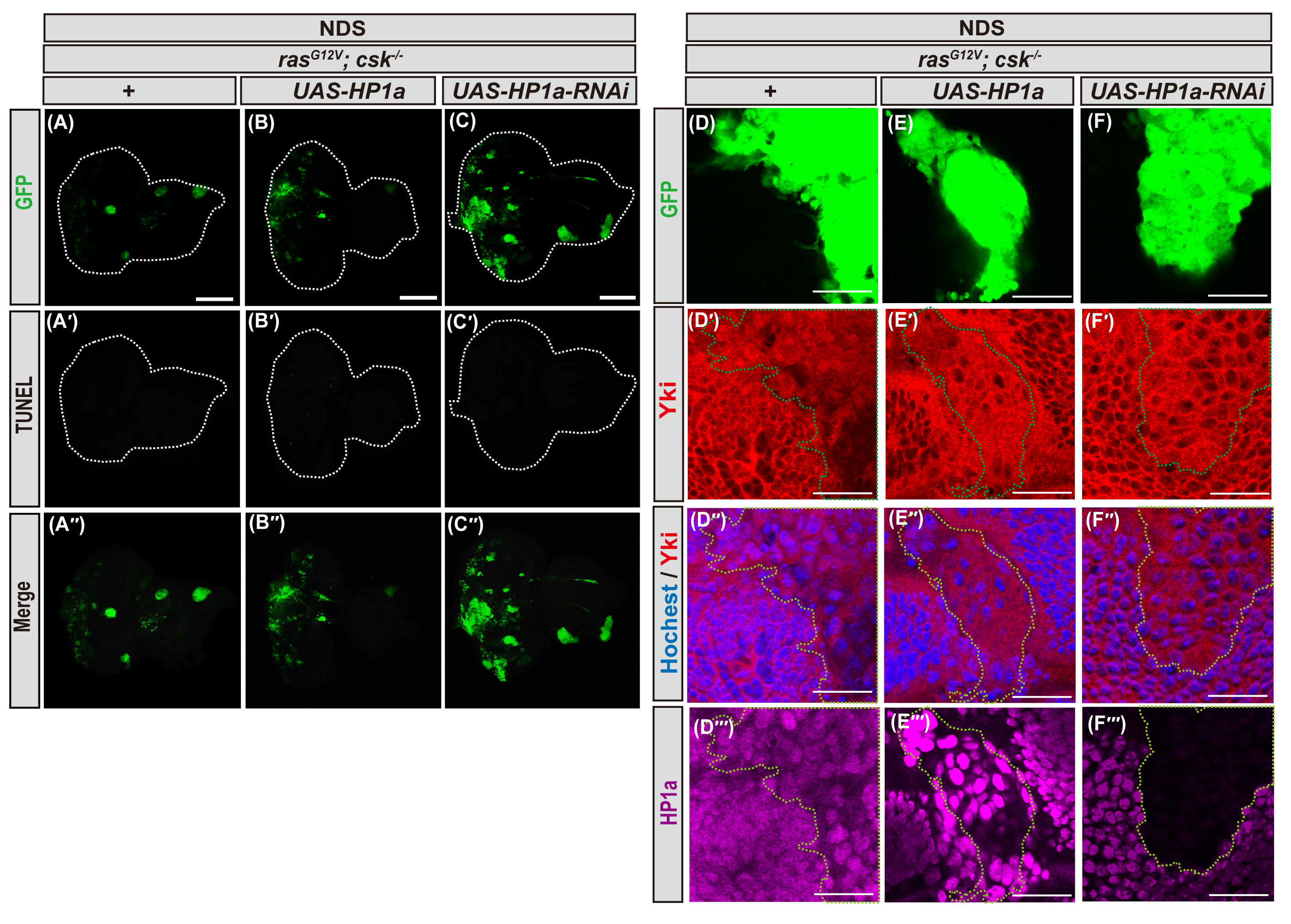

Supplement: Supplementary file 5 — Supplement Figure 4 [file 41419_2021_4414_MOESM5_ESM.tif]

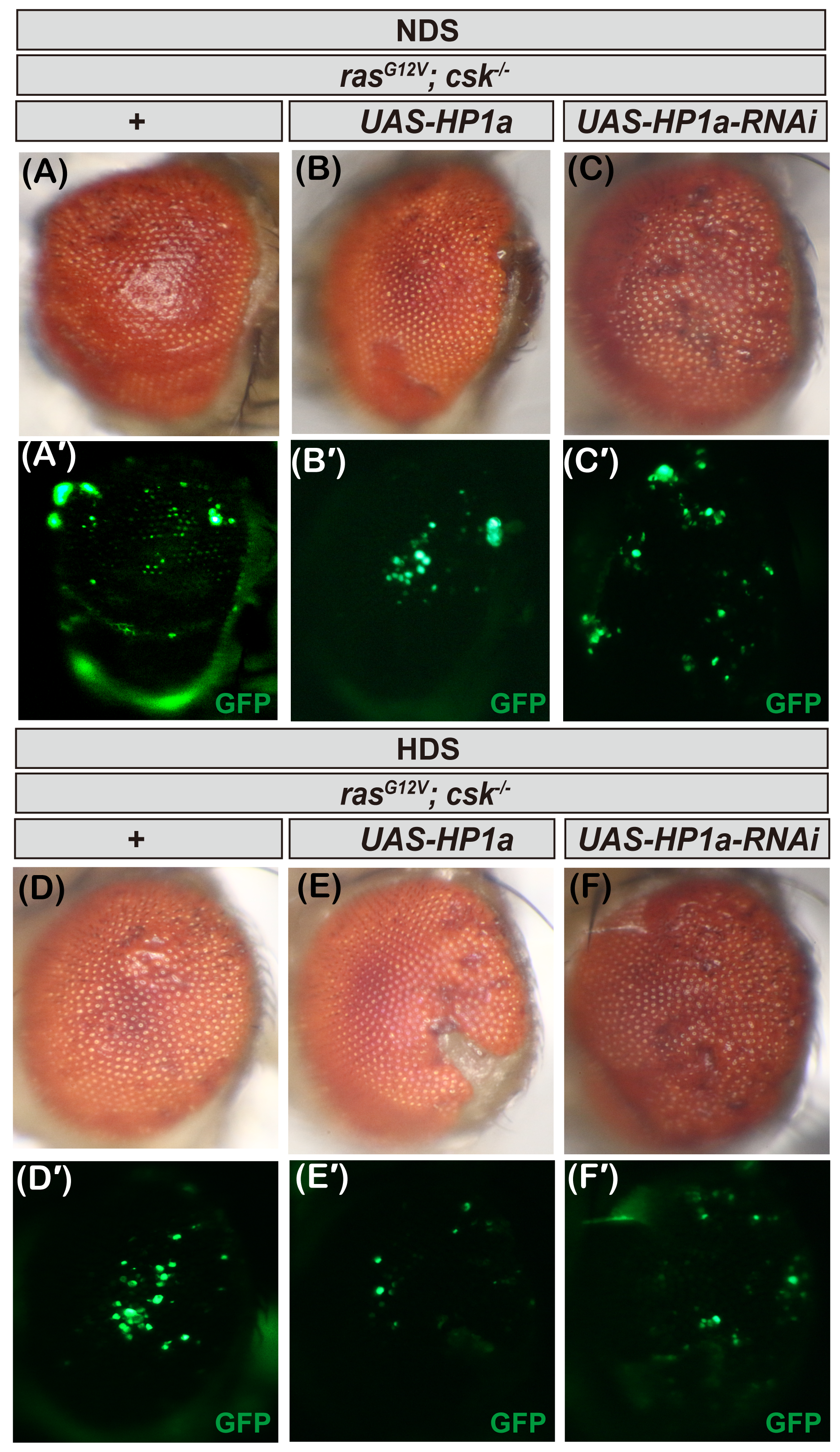

Supplement: Supplementary file 6 — Supplement Figure 5 [file 41419_2021_4414_MOESM6_ESM.tif]

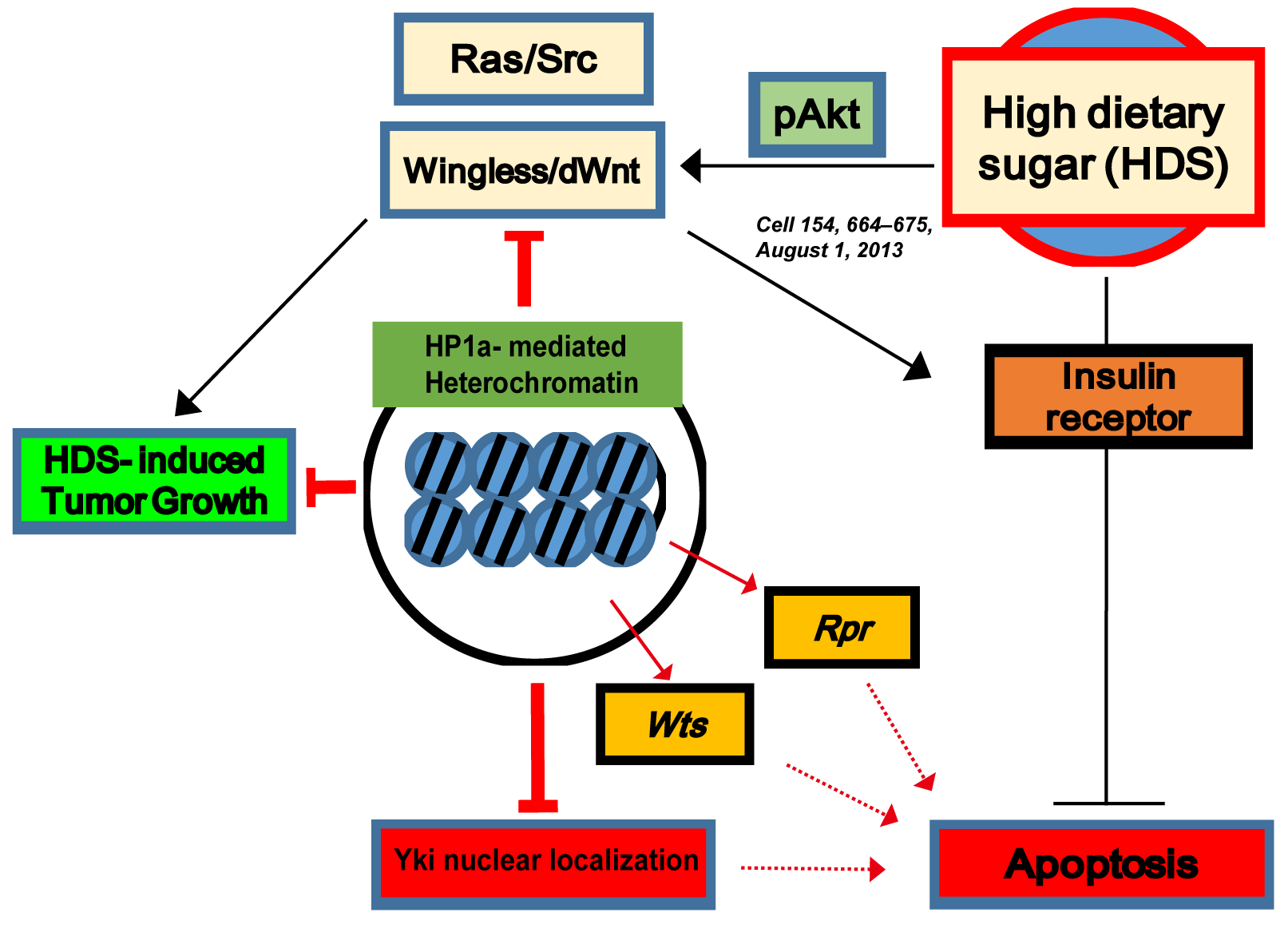

Supplement: Supplementary file 7 — Supplement Figure 6 [file 41419_2021_4414_MOESM7_ESM.tif]
